# MILO Mobile: An iPad App to Measure Search Performance in Multi-Target Sequences

**DOI:** 10.1177/2041669520932587

**Published:** 2020-06-20

**Authors:** Ian M. Thornton, Todd S. Horowitz

**Affiliations:** Department of Cognitive Science, Faculty of Media and Knowledge Sciences, University of Malta; National Cancer Institute, National Institutes of Health, Bethesda, Maryland, United States

**Keywords:** attention, visual search, action, memory

## Abstract

This article introduces a mobile app version of the Multi-Item Localization (MILO) task. The MILO task was designed to explore the temporal context of search through a sequence and has proven useful in both basic and applied research settings. Here, we describe the basic features of the app and how it can be obtained, installed, and modified. We also provide example data files and present two new sets of empirical data to verify that previous findings concerning prospective planning and retrospective memory (i.e., inhibitory tagging) are reproducible with the app. We conclude by discussing ongoing studies and future modifications that illustrate the flexibility and potential of the MILO Mobile app.

The Multi-Item Localization (MILO) task was developed as a computer-based tool for exploring the temporal context of visual search (Horowitz & Thornton, 2008; Thornton & Horowitz, 2004). On each trial, participants are required to search for a specific sequence of targets, such as the letters A through H, or the numbers 1 through 8. Participants respond by clicking on targets with a mouse or touching them on a touchscreen. The task can be tuned to explore either *retrospective* (i.e., the influence of previous actions on the localization of the current target) or *prospective* (i.e., the influence of future plans on the current target) aspects of search. The goal of this article is to introduce MILO Mobile, a freely available iPad version of the task, which we hope will encourage other researchers and clinical colleagues to further explore sequential search.

Our previous research with the computer-based version of the task identified several findings of interest (Horowitz & Thornton, 2008; Thornton & Horowitz, 2004). First, by introducing a manipulation in which detected targets either vanished or remained visible once selected, we were able to show that participants had almost perfect memory for locations they had already visited. Specifically, the serial reaction time (SRT) curves for the Vanish and Remain conditions had an almost identical shape (Thornton & Horowitz, 2004). Second, we showed that this memory was location rather than object-based, as the identity between the two SRT curves was broken as soon as either local or global motion was added to the displays (Horowitz & Thornton, 2008). Third, we demonstrated that participants consistently plan ahead when engaged in sequential search. We explored this by randomly shuffling the locations of targets ahead of the current target. While prospective effects are most obvious during search for the first item in a sequence, leading to a highly elevated response time to the first target, they are also manifest throughout the entire sequence, with planning occurring up to four items ahead of the current target (Thornton & Horowitz, 2004; see Kosovicheva et al., 2020 for related findings).

Historically, most visual search experiments involve displays with a single target. This design has allowed researchers to study the nature of search guidance, search templates, eye movement strategies, and so forth (see Hulleman & Olivers, 2017; Kristjánsson & Egeth, 2019; Wolfe & Horowitz, 2017 for recent reviews). Recently, a number of groups have also begun to explore more complex scenarios involving search for multiple target items (Cain et al., 2012; Fougnie et al., 2015; Gilchrist et al., 2001; Hills et al., 2012, 2013; Klein & MacInnes, 1999). Many of these studies were directly inspired by the animal foraging literature (Bond, 1983; Dawkins, 1971; Heinrich et al., 1977; Jackson & Li, 2004; Pietrewicz & Kamil, 1979; Tinbergen, 1960), and there appears to be a general feeling that extending search beyond a single target will help us better understand underlying attentional mechanisms.

What continues to make the MILO task distinctive is the fact that it involves *sequential* search. The new generation of multi-target tasks just mentioned are typically variants of nulling or cancellation paradigms (e.g., Dalmaijer et al., 2015; A. J. Woods et al., 2013), where items can be detected/cancelled in any order. The use of sequential search is a common component of neuropsychology assessment in the form of the Trail-Making Test (TMT; Reitan, 1958; for recent variants and discussion, see Fellows et al., 2017; Salthouse, 2011; D. L. Woods et al., 2015). However, the typical dependent measure in a clinical setting is overall completion time, and we believe the richer temporal context afforded by sequential search has yet to be fully exploited. With the MILO Mobile app, which can provide precise details of when (and where) each target in the sequence is located, we hope to encourage further exploration of sequential search.

The remainder of this article is organized as follows: We begin with a general description of the MILO Mobile app and how it can be obtained, installed, and modified. Next, we present two experiments that replicate and extend our previous computer-based studies, relating to retrospective and prospective effects during search through a sequence. Finally, we discuss a range of application scenarios and future directions.

## The MILO Mobile App

### Trial Display

**Figure 1.**
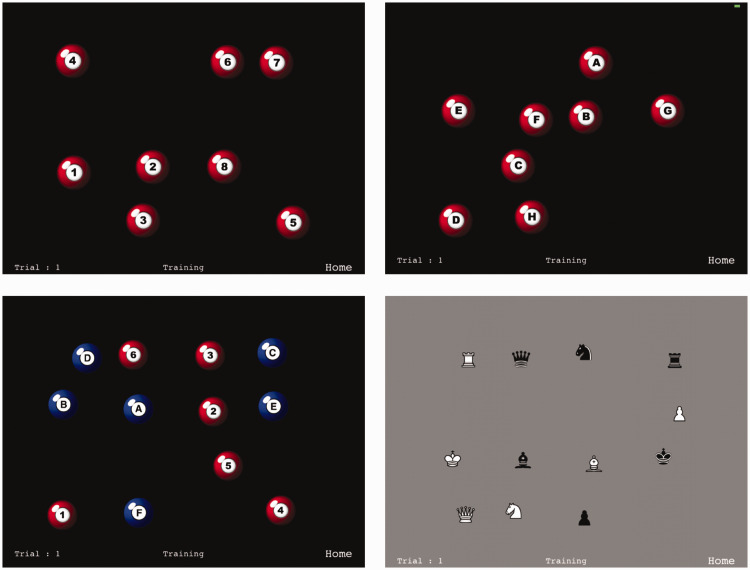
Example Screen Shots From the MILO Mobile App. Top row shows the digit and letter conditions used in Experiment 1. Bottom left shows a task variant in which the two types of sequences are interleaved within the same trial, making it possible to examine task switching. Bottom right, a task variant using a novel type of sequence, the precedence order of chess pieces.

Figure 1 shows example trials from the MILO Mobile app. In the studies reported in the current article, trial sequences were always eight items in length and consisted of either the letters A to H or the digits 1 to 8, as shown in the top row of the figure. The display can easily be modified to include more total items, more sequences and/or different sequences types. For example, in the lower left panel, each trial presents 2 sequences of 6 items, requiring 12 responses per trial. Depending on the app settings, participants can be asked to search through the two sequences consecutively, digits before letters (and vice versa), or the sequences could be interleaved, so that initial response sequences might be A, 1, B, 2, and so on (as in TMT version B).

The final panel in Figure 1 illustrates the ability to search through any type of sequences, here, the ordinal value of chess pieces (Zammit, 2017). In the current iPad version of the app, the addition of novel sequences requires a simple change to the code. In later versions of the app that are currently in development, sequence selection will be achieved simply by loading images of the appropriate size and specifying the response order.

### Task

Regardless of the nature of the sequence, the MILO task is always the same: select each item in turn as quickly as possible, starting at the first item in a sequence and ending with the last. In the MILO Mobile app, selection takes the form of directly touching one of the items with a finger. In the work reported later, and in our previous collaborative work (Basoudan et al., 2019; Jenkins et al., 2016, 2019) we have always specified that participants respond with the index finger of their dominate hand. Of course, any other instructions may be given, including the use of another finger, a stylus or even responding with more than one finger.

Although participants are asked to select items as quickly as possible, there is no time limit imposed. A trial ends either when the last item is selected or when an error occurs. An error only occurs if an item is selected out of sequence. When this happens, an error screen is displayed and a delay is introduced before the start of the next trial. In our work, such errors are extremely rare, and error rates have not proven to be a useful dependent variable. However, with some types of display and/or patient population combinations, error patterns may be of interest.

### App Settings

**Figure 2.**
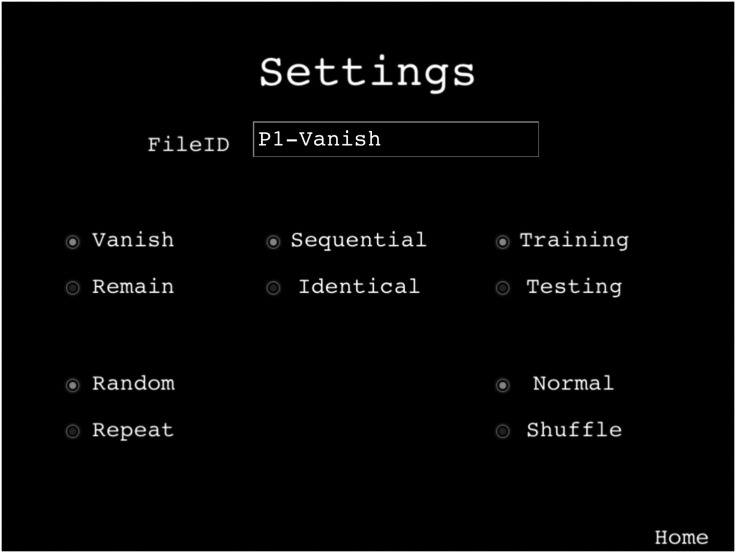
MILO Mobile Settings. A simple interface for setting task parameters. Each data file has a unique time code but can also be labelled with a user-defined text label. Radio buttons, from top left to bottom right, set the following parameters: Whether touched items vanish (default) or remain on the screen; whether the target items contain a sequence (default) or are identical, making it possible to study simple cancellation performance (see text for details); data are recorded when testing, but not when training (default); whether items values ahead of the current target shuffle or are fixed throughout the trial (normal, default).

The app settings page gives the researcher the ability to control the display and the experimental design (see Figure 2). In the experiments reported later, the manipulations we employed were selected via this page. For example, by default, items vanish once they have been selected. By changing the setting of a radio button, they can be made to remain on the screen, implementing the main retrospective manipulation we described earlier (see also Experiment 1). Similarly, by default, items ahead of the current target are unaffected once that target is selected. By selecting the Shuffle button in the settings page, the positions of items ahead of the current target can be randomized (see Experiment 2). More general experimental parameters such as the nature of the target items, file/participant id codes, and whether data are to be recorded are also managed through this interface.

### Data Capture and Analysis

When data recording is turned on, the app records the time and position of each selection event, and whether or not an error occurred. These data are written to standard text files, along with all other parameter settings for a given trial, and can be easily extracted from the device for further processing. Example output files and descriptive notes are provided on the OSF page associated with this article at https://osf.io/6bge9/.

In the work reported here, as in our previous studies, the main dependent measure is the SRT, the time elapsed since the last event (Horowitz & Thornton, 2008; Thornton & Horowitz, 2004). For the first target, this is the time from display onset. For all other targets, this is the time since the previous item was selected. As described in more detail in the experimental sections later, the pattern of SRTs across the full sequence can provide a detailed picture of how temporal context affects search for the current target item.

In future versions of MILO Mobile, we hope to implement in-app data visualization and analysis. This would be particularly useful in the context of clinical work, where, for example, only an immediate estimate of overall response variability or the cost of switching between one sequence and another might be required.

### App Availability and Modification

We initially decided to implement MILO Mobile as an iPad app as these devices—and the underlying iOS operating system—appeared to give rise to smaller and less variable timing errors than other available tablets (Burke et al., 2017; Pronk et al., 2019; Schatz et al., 2015). While there exist a number of useful benchmark studies comparing experimental platforms running on traditional computers (see Bridges et al., 2020 for review and up-to-date benchmarks), there is still only limited information available for mobile-based platforms. A review of the available material on mobile platforms, presented as a Supplementary Analysis, suggests that both native and web-based iPad applications are capable of providing sufficient temporal precision for use in chronometric studies. We also provide a direct comparison between iPad and desktop versions of MILO and a related task, finding in both that the tablet version gives rise to faster and less variable response times (https://osf.io/6bge9/).

At the time of writing, we are making the MILO Mobile app directly available to other researchers via Apple’s ad hoc distribution pipeline. To obtain a copy of the iPad app used for the current studies, simply send an email to the first author specifying the iPad model, Unique Device ID, and iOS version of the device you intend to use. These parameters are all displayed on *Summary* tab of iTunes when the device is connected to a computer. Alt-clicking the serial number will reveal the Unique Device ID and allow it to be directly copied. Once we receive this information, we can build a version of the app for your device and send it to you directly.

In addition, the Xcode project and all source files are being made available under the GNU3 General Public License (https://www.gnu.org/licenses/). These can be downloaded from the OSF page associated with this article at https://osf.io/6bge9/. This makes it possible for other groups to directly deploy the app to their devices as well as to modify the task to explore new research questions. We will also use the OSF repository to publish updates about the status of app and future cross-platform and/or downloadable versions. We are currently developing both a cross-platform version of MILO Mobile and an online version (https://maltacogsci.org/MILO/DEMO/), which we hope to make available shortly.

## Experiment 1

In Experiment 1, we wanted to verify that the MILO Mobile app could be used to record similar response patterns to those seen with the desktop version of the task in our previous studies (Horowitz & Thornton, 2008; Thornton & Horowitz, 2004). In particular, we wanted to replicate the finding that Vanish and Remain produce identical SRT slopes. Our interpretation of this pattern was that participants in the Remain condition were able to ignore previously visited items as effectively as if they had been erased from the screen. This may implicate some form of *inhibitory tagging* (Klein, 1988) of spatial locations (Horowitz & Thornton, 2008; Thornton & Horowitz, 2004).

The most obvious change, relative to our previous experiments, involved the use of the iPad to display stimuli and collect responses. In the original studies, the task was run on desktop computers and stimuli were presented on a standard monitor. Responses were collected via mouse clicks. In the current version of the task, stimuli were presented directly on the tablet screen and participants responded by touching targets with the index finger of their dominant hand.

However, several other modifications should be noted. In our original studies, we had used a set size of eight items, comprising a four-letter target sequence and a four-letter distractor sequence. On each trial, prior to the response display, a cue screen was presented showing the four targets for that trial. We did this for two reasons. First, to match target–distractor distinction typically seen in visual search tasks. Second, to avoid a potential accelerating response function in the Vanish condition; if there were no distractors, the final target would have a set size of one, whereas the final target in the Remain condition would have a set size equal to the number of targets. We were initially concerned this would confound our results.

For the iPad version, we wanted to explore a longer target sequence of eight items. Eliminating distractors simplified presentation and reduced overall running time. Furthermore, pilot testing indicated that the Vanish conditions did not show a differential acceleration relative to the Remain condition.

In all of our previous experiments, we had run separate blocks of Vanish and Remain trials, counterbalancing the order across participants. This was done to reduce noise related to switching between stimuli and decision sets. Here, we randomly interleaved Vanish and Remain trials to explore whether the condition blocking was a crucial factor in obtaining similar response functions.

Finally, in our previous experiments, we had exclusively used alphabetic sequences. Here, we had half of the participants search through a sequence of the letters A to H, and half of the participants search through a sequence of the digits 1 to 8 (see Figure 1). The goal here was to explore whether obtaining the same SRT pattern across conditions generalized to other types of sequences.

### Methods

#### Participants

A total of 24 participants (13 females; mean age = 20.8 years, *SD* = 1.4) from the Swansea University community took part in this study on a voluntary basis. Participants were randomly assigned to either the digit or alphabet groups, with 12 participants per group. Sample size was determined prior to data collection and was chosen to match the individual group sizes used in our previous studies. To verify that this sample size would provide adequate statistical power to capture changes in SRT as a function of target position, we computed the effect sizes for main effects involving this independent variable in our previous papers. Across 12 separate analyses, the average observed effect size (partial-eta squared) was 0.72 (*SD* = 0.2), with a range between 0.3 and 0.96. Using the lower end of this range to provide a conservative estimate, we conducted a prior power analysis using G*Power (Faul et al., 2007). This suggested a minimum sample size of nine participants per group, assuming required power of 0.95, an alpha level of .05, and minimal correlation (0.1) between the repeated measures.

All participants were right-handed and reported normal or corrected to normal vision. Prior to taking part in the study, all participants were given written information about the study, and consent forms which were signed. The study was designed and conducted with reference to the Helsinki convention and all aspects were reviewed and approved by the Swansea University Psychology Department Ethics Committee.

#### Equipment

The experiment was conducted using a first-generation iPad for both stimulus presentation and response collection. The iPad had a screen dimension of 20 × 15 cm and a resolution of 1024 × 768 pixels. Participants held the iPad (in landscape orientation) using their left arm and were required to respond using the index finger of their right hand. While viewing distance was not fixed, we estimated that it was approximately 50 cm from screen surface to the eyes. The MILO Mobile app was custom written in objective-C using Xcode and Cocos2d libraries. Source code is available on the OSF page associated with this article at https://osf.io/6bge9/.

#### Stimuli

The stimuli are shown in the top row of Figure 1. One group of participants saw the letter sequence A to H, while the other group saw the digit sequence 1 to 8. Characters were drawn in the context of red and white pool balls, which had shading to provide a slight 3D effect. Each ball had a diameter of 98 pixels and subtended approximately 2° visual angle. On each trial, the eight targets were randomly positioned within an invisible 4 × 4 grid that was centred on the screen with a 200 pixel (horizontal) and 150 pixel (vertical) offset from top left. The position of individual items within a grid slot was randomly jittered by up to 80 pixels horizontally and 30 pixels vertically.

#### Task and Procedure

The experiment was run in a quiet environment under low lighting conditions with no overhead lights, in order to minimize screen glare. Participants were first familiarized with holding the iPad and were shown the basic task of touching each target in sequence as quickly as possible. The correct response sequence was demonstrated as well as the method of response. As the Vanish and Remain trials were interleaved, this variation was explicitly shown. Participants were then allowed to practice responding to a series of training trails, including both conditions, until they were comfortable with the task. Typically, this training session lasted for 5 minutes. No data were recorded during this training phase.

The experimental phase of the task lasted approximately 15 minutes. Participants completed 2 blocks of 20 correct trials, each containing 10 Vanish and 10 Remain trials for a total of 40 trials. Error trials, which were extremely rare (<1% of total), were automatically replaced by immediately repeating a trial from the same condition, using a new sequence layout.

#### Data Analysis

Our main dependent variable was the SRT. For each participant, we calculated the median SRT at each target position over the 20 trial repetitions per condition. We use the median as our point estimate of the underlying SRT distributions, rather than a normalized/correct mean, as it is thought to provide a least biased estimate (see Rousselet & Wilcox, 2019 for a recent discussion). Median SRTs were analysed using a 2 (Condition) × 2 (Sequence) × 8 (Target) mixed analysis of variance (ANOVA) with Condition and Target as repeated measures and Sequence as a between subject factor. As our previous work led us to expect very different response times for the initial target (T1), compared with subsequent targets (T2–T8), we also conducted separate follow-up analyses. T1 responses were analysed using a 2 (Condition) × 2 (Sequence) type mixed ANOVA and T2 to T8 responses were analysed using a 2 (Condition) × 2 (Sequence) × 7 (Target) mixed ANOVA. Violations of the sphericity assumption involving the Target factor were corrected by applying the Greenhouse–Geisser adjustments to the appropriate degrees of freedom.

Full details of all statistical tests can be found in Supplemental Table 1 and raw data have been uploaded to the OSF page associated with this article at https://osf.io/6bge9. In addition to our null hypothesis tests, we also conducted Bayesian analysis. Specifically, we used the approach outlined by Masson (2011) to estimate the weight of evidence in favour of each of our main effects and interactions. In the text, we report both the Bayes Factor (BF) and related posterior probability (e.g., p(H_0_|D)), for the sake of completeness. Where only weak evidence was obtained, defined as, BF <3.0 and posterior probability <.75 (Jeffreys, 1998; Raftery, 1995; Wagenmakers et al., 2011), this is noted in the text.

### Results

**Figure 3.**
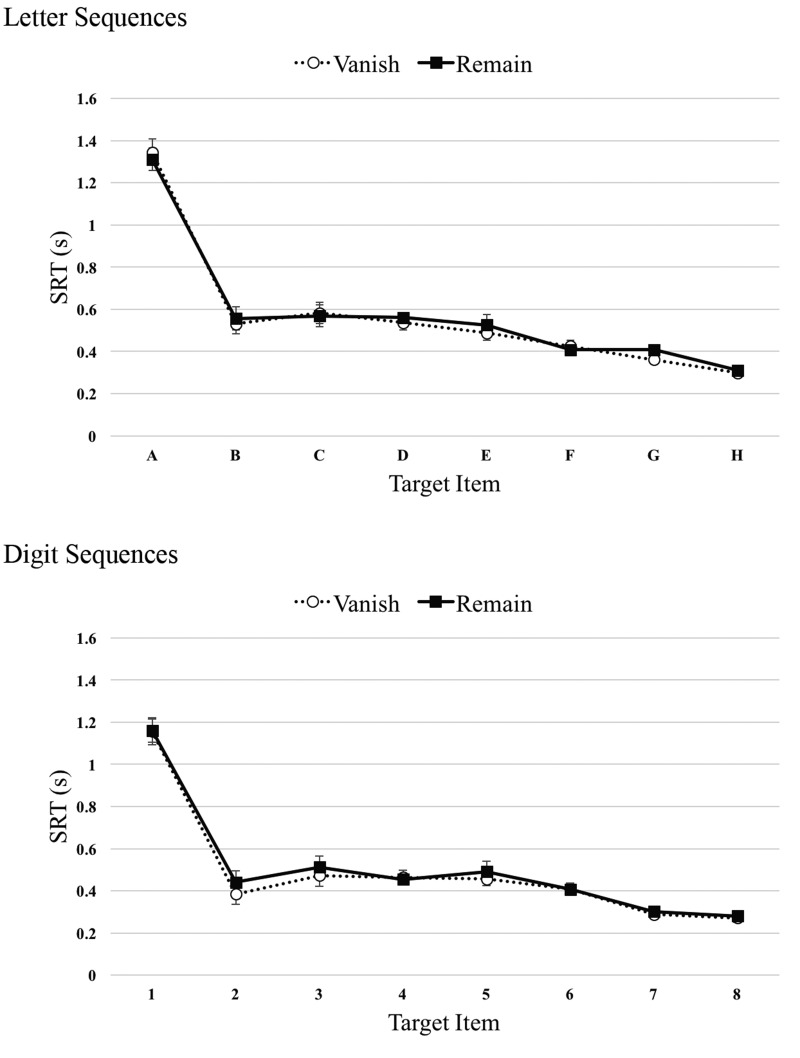
Results of Experiment 1. Top panel: Median SRT patterns for both Vanish and Remain conditions as a function of target letter (A–H). Lower panel: Median SRT patterns for both Vanish and Remain conditions as a function of target digit (1–8). Error bars represent 1 standard error of the mean. SRT = serial reaction time.

Figure 3 shows the SRT × Target patterns for the Vanish and Remain conditions of Experiment 1, with letter and digit groups presented in separate panels. The overall pattern replicates our previous findings. T1 responses were elevated relative to the T2 to T8 responses. Following a 2 or 3 item plateau, the curve accelerates quite quickly. The most striking feature of the data is the clear overlap between the Vanish and Remain functions, again replicating our previous findings.

Statistically, there was a main effect of Target, *F*(3.3,72.1) = 200, MSE = 0.02, *p* <.001, $\eta_{p}^{2}\;$= 0.90, BF_10_ >100, p(H_1_|D) >0.99, but no interactions involving Target and any other factor (see Supplemental Table 1, all BF_01_ >3). There was a main effect of Condition, with slightly faster average responses during Vanish (*M* = 529 ms, *SE* = 18) than Remain (*M* = 544 ms, *SE* = 19) trials, *F*(1,22) = 6.8, MSE = 0.003, *p* <.05, $\eta_{p}^{2}\;$= 0.24, BF_10_ = 5.0, p(H_1_|D) = 0.84. There was also a main effect of Sequence, with faster responses for digit sequences (*M* = 497 ms, *SE* = 26) compared with alphabetic sequences (*M* = 576 ms, *SE* = 26) trials, *F*(1,22) = 4.7, MSE = 0.13, *p* <.05, $\eta_{p}^{2}\;$= 0.18, BF_10_ = 2.1, p(H_1_|D) = 0.67. We note, however, that the evidence supporting this effect is relatively weak. The Condition × Sequence interaction was not significant (see Supplemental Table 1).

Analysis of the T1 responses showed only a significant main effect of Sequence, with initial responses to the digit sequences (*M* = 1,159 ms, *SE* = 57) tending to occur more quickly than responses to the letter sequences (*M* = 1,328 ms, *SE* = 57), *F*(1,22) = 4.4, MSE = .08, *p* <.5, $\eta_{p}^{2}\;$= 0.17, BF_10_ = 1.8, p(H_1_|D) = 0.65. Again, we note that the evidence supporting this effect is relatively weak. No other effects were significant (see Supplemental Table 1).

Separate analysis the T2 to T8 responses revealed a main effect of Target, *F*(3.3,72.4) = 28.8, MSE = 0.01, *p* < .001, $\eta_{p}^{2}\;$= 0.57, BF_10_ >100, p(H_1_|D) >0.99, but no hint of any interactions, all BF_01_ >3. The nonlinear decrease in SRTs as a function of target item gave rise to significant linear, quadratic, and cubic polynomial trends (all *F*s >10, *p*s <.01, $\eta_{p}^{2}$s >0.3). The only other significant T2 to T8 effect was a main effect of Condition, *F*(1,22) = 10.4, MSE = 0.003, *p* <.05, $\eta_{p}^{2}\;$= 0.32, BF_10_ = 21.1, p(H_1_|D) = 0.96, again reflecting slightly faster average responses during Vanish (*M* = 426 ms, *SE* = 19) than Remain (*M* = 445 ms, *SE* = 19) trials.

### Discussion

The results of Experiment 1 closely replicate the main SRT patterns observed in our previous studies (Horowitz & Thornton, 2008; Thornton & Horowitz, 2004). Specifically, T1 responses were clearly elevated relative to all other responses, and the shape of the Vanish and Remain patterns did not differ as a function of target position. This confirms that the MILO Mobile app performs as expected and can be used in addition to or in place of the original desktop task for exploring the temporal context of search.

As well as closely replicating our previous work, the current experiment adds to our existing knowledge in two important respects. First, we have shown that nature of the sequence—letters versus digits—has relatively little impact on the overall Target × Condition search function. While there was a slight slowing when searching through letters versus digits, this seems to be mostly driven by the T1 responses, which may simply reflect the simplicity and salience of an initial “1” versus an initial “A” target. In any event, the current data only provided relatively weak evidence in favour of the Sequence main effect in both of our analyses, suggesting some caution in interpreting this finding.

More generally, and as discussed in more detail in the final section of the article, in other work we have begun to explore very different types of target sequence—lightness patches and chess pieces, for example—and have also been able to replicate the main SRT patterns discussed earlier. Thus, we can be fairly confident in suggesting that the elevated first response and the ability to tag previously visited locations (i.e., identity between Vanish and Remain patterns) are general characteristics of searching through a sequence and are not specific to particular stimulus sets.

The second novel finding from Experiment 1 is that the overall shape of the Vanish and Remain search functions is still the same even when these two types of trial are randomly interleaved within a block. In all of our previous studies, this factor had been blocked to reduce potential measurement noise. The current findings suggest that the ability to tag past locations is relatively robust, although as we note shortly, there may be some overall reaction time cost associated with switching task from trial to trial. From a practical point of view, the ability to interleave trial types has the potential to simplify and shorten assessment within a clinical setting. Very often, a battery of tests is administered to patients, and thus a single session of MILO trials reduces the overall complexity of the session and avoids the issue of block order effects.

Although there was no Target × Condition interaction in the current experiment, there was a small (<20 ms) overall speed disadvantage for Remain trials. We have observed such a constant offset in a previous experiment, albeit in the context of the Shuffle manipulation (Thornton & Horowitz, 2004; Experiment 2). There, we suggested the slowing in Remain trials could reflect additional cognitive load associated with *tagging* old targets (Watson & Humphreys, 1997) or some form of generalized masking due to the constant visual clutter when items do not vanish. Another possibility in the current experiment could arise due to the interleaving of the Vanish and Remain trials. As participants are not aware of the nature of a trial until they respond to the first target—note there was no effect of Condition for T1 responses—its failure to vanish could conceivably cause some short-lived disruption or surprise-related slowing that is present from T2 onwards. Again, as the offset appears constant and does not interact with sequence position, the more important finding is that search appears to proceed in a very similar fashion regardless of whether items vanish or remain visible.

## Experiment 2

In Experiment 2, we shift the focus to the third main finding from our previous work, the idea that observers consistently plan ahead when searching through a sequence (Horowitz & Thornton, 2008; Thornton & Horowitz, 2004). The clearest indication of this behaviour can be seen in elevated T1 responses compared with T2 to T8 responses. Here, we wanted to replicate the finding that shuffling the identity of items ahead of the current response target largely eliminates the difference between T1 and all other responses. Ten participants completed 2 blocks of Vanish trials, 1 block identical to the digit condition of Experiment 1, the other involving the Shuffle manipulation, described in more detail later.

### Methods

#### Participants

Ten participants (5 females; mean age = 30.3 years, *SD* = 2.2) from the Swansea University community took part in this study on a voluntary basis. The sample size was determined and verified as per Experiment 1. All participants were right-handed and reported normal or corrected to normal vision. Prior to taking part, all participants were given written information about the study and consent forms, which were signed. The study was designed and conducted with reference to the Helsinki convention and all aspects were reviewed and approved by the Swansea Psychology Department Ethics Committee.

#### Equipment and Stimuli

These were identical to those used in Experiment 1, with the exception that only the digit sequence 1 to 8 was used.

#### Task and Procedure

These were the same as those used in Experiment 1, with the following exceptions. In a first block of experimental trials, participants searched through the digit sequence 1 to 8, completing 20 correct trials of the Vanish condition. They were then introduced to the Shuffle manipulation. The sequence and target behaviour were the same as in the normal Vanish condition, but after each response, the identity of subsequent target items was shuffled within the existing locations. Thus, the objects did not change position, only the digits. Participants were given the opportunity to practice with this version of the task until they were comfortable and then completed an experimental block of 20 correct trials. Note we did not counterbalance the order of the blocks, as our previous research had demonstrated that the Shuffle manipulation was considerably more demanding. Here, then, the initial block of 20 Vanish trials essentially serves as baseline for comparison with the Shuffle block.

#### Data Analysis

The only change relative to Experiment 1 was that we did not conduct separate analyses of the T1 and T2 to T8 data. Our previous studies gave a clear indication of the shape of the SRT function to be expected in the Shuffle condition.

### Results

**Figure 4.**
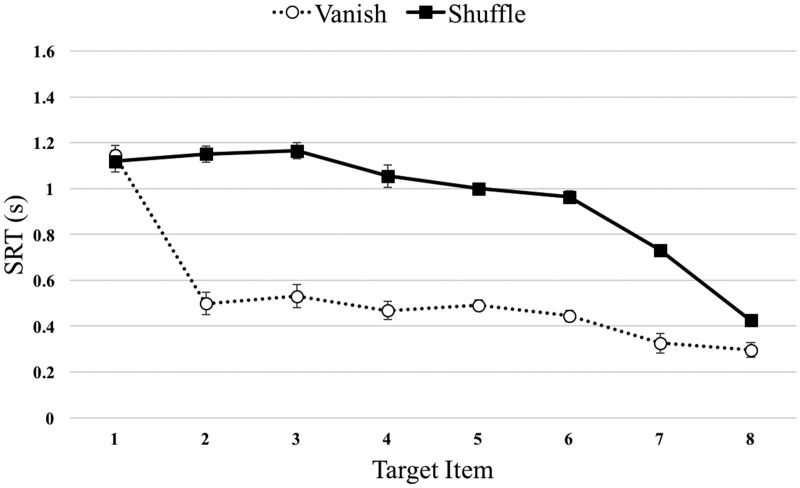
Results of Experiment 2. Median SRT patterns for both Vanish and Shuffle conditions as a function of target digit (1–8). Error bars represent 1 standard error of the mean. SRT = serial reaction time.

Figure 4 shows the SRT × Target patterns for the Vanish and Shuffle conditions of Experiment 2. The Vanish condition essentially replicates the pattern seen in our previous work and in Experiment 1. After the initial T1 response, the remaining targets are located much more rapidly with steadily decreasing SRTs as the sequence progresses. In contrast, T1 to T3 responses in the Shuffle condition remain at the initial elevated level, and even seem to increase slightly. As the set size physically decreases in later parts of the sequence SRTs reduce quite rapidly, but only approach those seen for the Vanish condition for the very final response.

There were significant main effects of both Condition, *F*(1,9) = 446.0, MSE = 0.016, *p* <.001, $\eta_{\text{p}}^{2}$ = 0.98, BF_10_ >100, p(H_1_|D) >0.99 and Target, *F*(3.04, 27.37) = 95.4, MSE = 0.025, *p* <.001, $\eta_{\text{p}}^{2}$ = 0.91, BF_10_ >100, p(H_1_|D) >0.99. However, these must be interpreted in the context of the very clear Condition × Target interaction, *F*(3.57, 32.15) = 33.5, MSE = .018, *p* <.001, $\eta_{\text{p}}^{2}$ = 0.79, BF_10_ >100, p(H_1_|D) >0.99.

### Discussion

As in our previous studies, the Shuffle manipulation changed the overall shape of the SRT function. Specifically, it eliminated the large difference between T1 responses and the later items in the sequence. Consistent with the results of Experiment 1, this again verifies that the MILO Mobile app can be used to explore the temporal context of search. More generally, the Shuffle manipulation provides a simple, yet powerful way to modulate the prospective aspects of searching through a sequence by blocking the ability to plan responses in advance (see also Kosovicheva et al., 2020). Previously, we have shown that such planning can occur up to four items ahead in a sequence and takes place during both Vanish and Remain trials (Thornton & Horowitz, 2004). In the next section, we discuss how additional manipulations can also shed light on prospective effects, in particular, elevated T1 responses.

## Summary and Future Directions

In this article, we have introduced a mobile app version of the MILO task. By making the app and the source code freely available, we hope to encourage others to further explore the temporal context of search behaviours, both in relation to basic research questions, and also as possible markers of individual and/or group differences. In two experiments, we used the app to replicate our previous findings that both the prospective and retrospective temporal context of a sequence influence ongoing search. Specifically, we used the Vanish/Remain manipulation to show that items that have already been found are essentially ignored at later stages of a trial, and the Shuffle manipulation to illustrate that participants plan ahead while responding to the current target.

In our own ongoing research, we are hoping to use the MILO Mobile app to further explore these two aspects of search context. In one series of studies, we have focused specifically on what causes the elevated first response (Tsui et al., 2013). Using identical target items that can be selected in any order (see Figure 2, *Identical* radio button), we have shown that a substantial proportion of the T1 slowing appears related either to the registration of display onset or initiation of the first motor action, rather than to sequence-specific search and action planning. That is, T1 responses to identical targets remain constant at approximately 600 ms when set size is randomly varied between one and six items from trial to trial, while T2 to TN responses do not vary and are all completed in around 200 ms. For numeric sequences, the T1 responses rise linearly from the initial 600 ms level at set size of 1, increasing by approximately 60 ms/item as set size increases to reach the levels found in Experiments 1 and 2 in the current work. T2 to TN responses follow the SRT patterns described earlier.

A second study in this series (Tsui et al., 2013), points more strongly to the idea that the constant component of the T1 delay is related to motor initiation. Across different blocks of trials, we had participants wait a fixed interval while *previewing* the fully visible search array containing eight items. After the preview period, which could be 0, 2, 4, or 6 seconds, the screen border changed colour and responses could begin. The T1 responses were always elevated, relative to T2 to T8 responses, and dropped from around 1.4 seconds with zero delay to approximately 750 ms with a 6-second delay. The persistence of the elevated T1 response, even without the demands of registering a new visual layout, would seem to implicate differences in motor fluency between the first and subsequent motor actions in a sequence.

In other work, we have begun to explore more fully whether the nature of the target sequence affects the ability to retrospectively *tag* previous locations. When searching through sequences of achromatic patches varying in lightness from white to black, or vice versa (Zdravkovic et al., 2012) or through sequences defined in terms of the precedence order of chess pieces (Zammit, 2017), the overall SRT patterns are very similar to those reported here. More specifically, we again found no interaction between the Vanish/Remain condition and target position with either of these types of sequence, suggesting that location tagging occurs in a variety of settings.

More recently, we have begun to explore SRT patterns when the display consists of two different sequences (Thornton & Horowitz, 2020). For example, in the display shown in, Figure 1, lower left panel, participants can be asked to cancel all letters followed by all digits (or vice versa) in a 12-item sequence. In this *sequential* condition, SRT patterns are very similar to those observed with a single sequence. T1 responses are always elevated and Vanish and Remain trials give rise to almost identical patterns of SRTs. The only difference from a typical MILO trial is a clear switch cost at T7, when the second sequence begins, giving rise to a slowing of approximately 360 ms.

The potentially more interesting condition is when the two sequences are interleaved within a trial, such that participants must respond A-1, B-2, and so on. This mimics the more demanding TMT-B condition, which is often used to probe for difficulties with Executive Control (e.g., Salthouse, 2011). Our initial results suggest two very interesting findings. First, the overlap between Vanish and Remain conditions is much reduced, with the slowing for Remain trials appearing to increase as the sequence progresses. The added demands of this condition may thus disrupt the ability to apply inhibitory tagging or marking (Klein, 1988; Klein & MacInnes, 1999; Wang & Klein, 2010; Watson & Humphreys, 1997). Second, both Vanish and Remain SRTs grouped in successive fast–slow pairs (A-1 and B-2) giving a distinctive saw-tooth pattern. This suggests that repeated working memory chunking, rather than alternate switching between entire sequences, may underlie the cognitive load associated with this sort of task. Importantly, both of these novel findings rely on examining responses to all items in the sequence, rather than simply measuring overall completion time, as is typically done with tasks such as the TMT.

Finally, although our primary focus has been on using MILO Mobile to explore the basic nature of sequential search, we also believe it has potential as a measure of individual differences in clinical/applied settings. In general, the use of computer-based and mobile testing is now becoming much more common in the context of neuropsychological assessment (Germine et al., 2019; Schatz et al., 2015). In our own work together with colleagues in the United Kingdom, we have already begun to use the MILO Mobile app to explore changes in response time patterns as a function of both normal (Basoudan et al., 2019; Jenkins et al., 2016) and abnormal (Jenkins et al., 2019; Richards, Thornton, Bayer, Norris, Hanley, Richardson, et al., 2020) aging. As in our basic research mentioned earlier, examining the pattern of response times across the entire sequence yields novel findings. For example, we have found that the variability associated with the initial response in a sequence can be used to successfully distinguish both healthy young from healthy older adults (Basoudan et al., 2019) and patients with vascular cognitive impairment from age-matched controls (Richards, Thornton, Bayer, Norris, Hanley, Richardson, et al., 2020). Furthermore, the slope of the Remain SRT function changes relative to the Vanish slope in vascular cognitive impairment patients, but not in age-matched controls (Richards, Thornton, Bayer, Norris, Hanley, & Tales, 2020). These initial results indicate both that the MILO Mobile app can be effectively used with patient groups, and that measures derived from SRT patterns are sensitive enough to identify clinically relevant individual and group differences.

## Supplemental Material

sj-pdf-1-ipe-10.1177_2041669520932587 - Supplemental material for MILO Mobile: An iPad App to Measure Search Performance in Multi-Target Sequences Click here for additional data file. Supplemental material, sj-pdf-1-ipe-10.1177_2041669520932587 for MILO Mobile: An iPad App to Measure Search Performance in Multi-Target Sequences by Ian M. Thornton and Todd S. Horowitz in i-Perception
