# Supplementary material for: MILO Mobile: An iPad App to Measure Search Performance in Multi-Target Sequences
Source: Iperception. 2020 Jun 20;11(3):2041669520932587. doi: 10.1177/2041669520932587 (PMC7307404; doi:10.1177/2041669520932587)
Supplement: sj-pdf-1-ipe-10.1177_2041669520932587 - Supplemental material for MILO Mobile: An iPad App to Measure Search Performance in Multi-Target Sequences [file sj-pdf-1-ipe-10.1177_2041669520932587.pdf]

SUPPLEMENTARY ANALYSIS for:

MILO Mobile: An iPad app to measure search performance in multi-target sequences.

Ian M. Thornton (1) & Todd S. Horowitz (2),

(1) Department of Cognitive Science, Faculty of Media and Knowledge Sciences, University of Malta, Msida, MSD 2080, Malta.
(2) National Cancer Institute, National Institutes of Health, Bethesda, MD 20892, USA

Correspondence and reprint requests can be made to Ian M. Thornton email: ianthornton@um.edu.mt

Comparing reaction time distributions on iPad versus desktop implementations of experimental tasks

Overview

The purpose of this analysis is to establish whether response times collected via an iPad are likely to reduce the sensitivity of the MILO Mobile app relative to previous desktop implementations. Of particular interest is any indication that iPad responses give rise to substantial delays or produce reaction time distributions that are inherently noisy, possibly obscuring measures of interest (Brand & Bradley, 2012; Miller et al., 2018; Plant, 2016; Plant & Quinlan, 2013; Reimers & Stewart, 2007; Ulrich & Giray, 1989; see Bridges et al., 2020 for recent discussion). While there exist a number of useful benchmark studies comparing experimental platforms running on traditional computers (see Bridges et al., 2020 for review and up-to-date benchmarks), there is still only limited information available for mobile-based platforms (although see Burke et al., 2017; Germine et al., 2019; Pronk et al., 2019; Schatz et al., 2015, discussed below). This is despite the growth in the use of mobile-based testing, particularly in the context of neuropsychological assessments (Germine et al., 2019; Schatz et al., 2015).

Here, we begin by reviewing what is known about iPad response time characteristics from previous studies. We then provide two analyses of MILO-related data, qualitative and quantitative, where we directly compare response times collected on desktop versions of a task, using the mouse as an input device, to iPad versions of the task, using the touchscreen.

General considerations when using iPads to collect RT data

We initially decided to implement MILO Mobile as an iPad app as these devices -- and the underlying iOS operating system -- give rise to smaller and less variable timing errors than other available tablets (Burke et al., 2017; Paul, 2013; Pronk et al., 2019; Schatz et al., 2015). One early estimate of the so-called Minimum App Response Time (MART; Agawi Touchmarks, reported in Paul, 2013) – the time from a touch on the screen to the initial display of a simple visual stimulus – suggested that iPads provided responses within 81 ms, compared to, for example, the Surface RT (90 ms), Kindle Fire HD (114 ms), Nexus 7 (135 ms) or Galaxy Tab 3 (168 ms). The MART, however, is not a very direct measure of response time, as it also includes the whole OS rendering pipeline (Luu, 2017).

Schatz et al., (2015) used a photosensitive mechanical actuator to more directly measure responses to the appearance of a simple visual stimulus. Their custom app running on an iPad could register touch responses with a constant delay of 27-33 ms, comparable with that expected from a standard USB keyboard (Neath et al., 2011). In addition to assessing the absolute lag or accuracy of responses, the authors also noted that iPads responses were precise, in the sense of giving rise to the same or less variability than any other device. We return to the issue of response variability shortly (see also Bridges et al., 2020 for further discussion of the accuracy versus precision distinction in this context).

In another study, Burke et al., (2017) compared human response times measured either via an iPad or using a traditional Visual Choice Reaction Time Apparatus (Lafayette Instrument Company, Lafayette, IN), a dedicated hardware device designed to precisely measure motor responses. For simple, single-touch tasks (comparable to MILO Mobile), they found no difference in either mean absolute reaction time (300 vs 287 ms) or in the SEM of those responses (7 vs 9 ms), between the traditional and iPad measures, respectively. Furthermore, the test-retest performance of the two types of devices did not differ.

The above studies suggest that iPad devices using dedicated apps are capable of recording responses with acceptable levels of absolute delay and precision. Furthermore, Pronk et al., (2019) recently evaluated response times from a range of mobile and desktop devices within the context of generic web applications, simulating simple app events using JavaScript. In controlled lab situations, iOS web applications (albeit running on an iPhone rather than an iPad) again gave rise to lower RT overestimations (approximately 60 ms) compared to any other device. More generally, the variability of responses across trials within touchscreen devices was also noted to be small and consistent (approximately 8 ms), leading the authors to conclude that "…touchscreen devices seem technically capable of administering a substantial number of mental chronometry paradigms, when taking some limitations and best practices into account" (p11).

While a specific device may have the capacity to accurately and reliably measures responses, it is also important to show that a given app running on the device in fact does so. As noted above, of particular importance in the current context, is whether an app can deliver precise timing of successive responses. As the main dependent measure for MILO is the pattern of Serial Reaction Times (SRT; see main text; Horowitz & Thornton, 2008; Thornton & Horowitz, 2004), this within-device precision is crucial. Generally, as long as the observed "technical" precision is within the limits of expected human trial-by-trial variability in response

times it should be possible to measure useful experimental effects (see Bridges et al., 2020 for further discussion).

In our previous work, we have used the same core software routines that control display and response collection in MILO Mobile to implement a number of other time-critical experimental tasks. As we have been able to measure meaningful changes in participant behavior across conditions in these studies (Basoudan et al., 2019; Á. Kristjánsson et al., 2014; Thornton et al., 2014; Thornton & Horowitz, 2015), this is a first indication that the device and app technical precision operate within acceptable limits.

However, to more directly examine the characteristics of responses obtained via the iPad, below, we present comparisons with two desktop studies in which the mouse was used as an input device, rather than the touchscreen. We begin by simply plotting data from the current Experiment 1 against the most comparable condition from our original MILO study (Thornton & Horowitz, 2004). This should provide a general indication of whether iPad responses introduce noticeable delays or increase the variability. To more precisely characterize response distributions with the two input modalities, we then present data for a MILO-related cancellation task, where task demands across modalities were more closely matched.

Comparison between original MILO and MILO Mobile SRT patterns

To make an initial assessment of the nature of iPad responses, we directly compared data from the main study of the current paper with those collected via a computer mouse in our original study (Thornton & Horowitz, 2004). As noted in the main text, in addition to the fact that the original data were collected on a desktop computer, there were several other subtle, but important, task differences that need to be borne in mind. Specifically, in the original study, Vanish and Remain conditions were blocked, rather than interleaved; displays contained eight items, but a novel target sequence of 4 items was pre-cued on each trial; search thus took place within the context of 4 distractor items. These differences clearly complicate the interpretation of differences between the two sets of data. Nevertheless, as our goal here is to establish whether iPads introduce noticeable lags in human response times and/or increased noise, such a comparison may still prove useful.

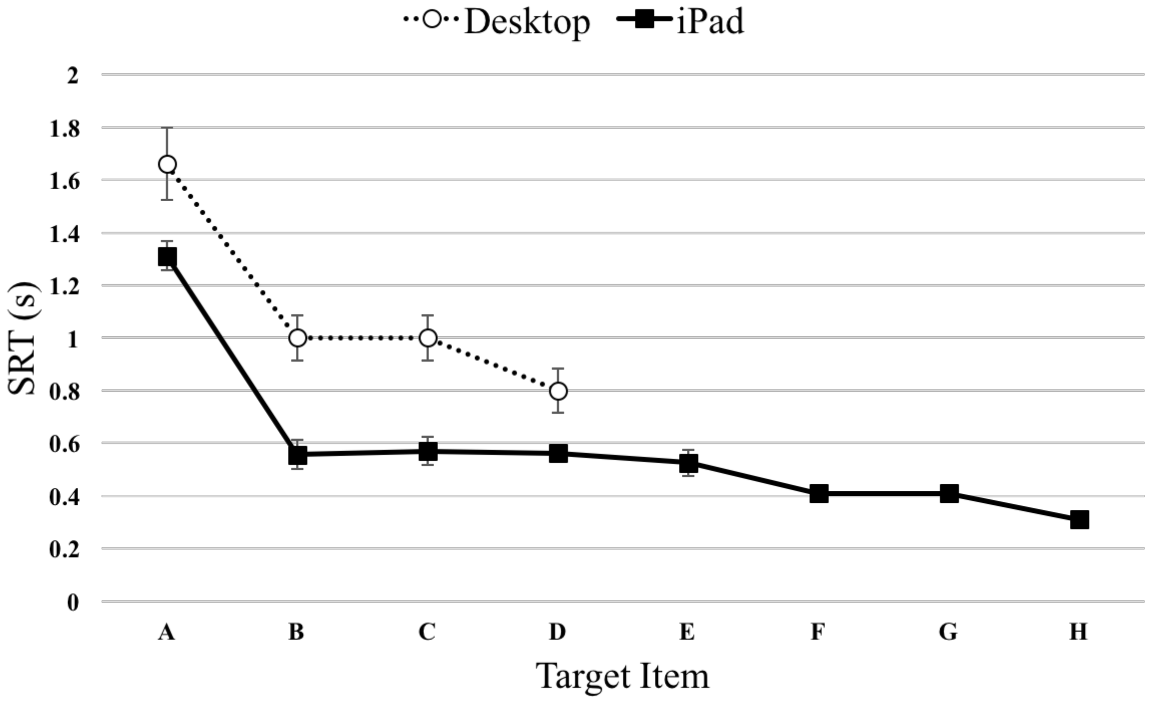

Figure S1. Comparison between Vanish performance from Thornton & Horowitz (2004; Experiment 1) and the iPad data from the letter sequences of Experiment 1 in the current paper. Error bars represent the standard error of the mean.

As can be seen in Figure S1, iPad responses were consistently faster that the equivalent Desktop responses. The between-subject variance captured in the error bars also appears to be considerably higher when responding with the mouse. This pattern appears consistent with data reported by Germine et al., (2019), where response times to an online version of the Trail

Making Test (Reitan, 1958; see main text) were 24-30% faster on an iPad compared to a Macintosh computer. The authors also note that the proportion of shared variance between version A and B of the task was 50-90% greater on the iPad.

Examination of RT data from a related foraging task.

While the previous comparison does not raise immediate concerns in relation to iPad responses, we also felt it would be useful to conduct a more quantitative assessment of mouse versus touchscreen reaction time distributions. To do this, we made use of data obtained using both iPad and desktop computer implementations of the exact same foraging cancellation task (Clarke et al., 2018; Á. Kristjánsson et al., 2014). This foraging task not only shares many of the same response characteristics as MILO, in that it requires multiple target responses per trial, but the iPad version of the task was implemented using the same core software routines used for MILO Mobile.

In both versions of the task, participants were required to make 40 rapid cancellation responses per trial. Here, we examined 10 trials from 64 participants in each response modality, providing > 50K responses for the comparison. Below, we describe the task and data sets in more detail, before directly comparing the response distributions.

Task Description

On each trial of the foraging task, participants were presented with a display containing 80 randomly distributed objects. The task was to locate and cancel 40 pre-defined target objects, while avoiding 40 distractor objects. In some blocks of trials, targets were distinguished by a single-color feature, while in other blocks targets were defined by a conjunction of color and shape. In the original, iPad version of the task (Á. Kristjánsson et al., 2014), targets were cancelled by directly touching them, as in MILO Mobile. In the desktop version of the task (Clarke et al., 2018), which was specifically designed to closely replicate the iPad study, targets were cancelled by clicking the mouse. Here, we only report data from Feature blocks, as this condition has been shown to give rise to rapid, temporally stable responses (T. Kristjánsson et al., 2020).

Equipment

As already noted, the iPad version of the foraging task was implemented using exactly the same display and response routines used for the MILO Mobile app (see main text). The desktop version of the task was implemented using the Psychophysics toolbox (Brainard, 1997; Kleiner et al., 2007; Pelli, 1997) in MATLAB, and was conducted on a PowerMac running OS X 10.8.2 connected to a 17-inch CRT monitor with a resolution of either 1400 x 1050 (n = 40) or 1600 x 1200 (n = 24) pixels (Clarke et al., 2018; A. Hunt, personal communication, 19 March 2020).

Date Sets

Data from 64 participants who completed the iPad version of the task was obtained from three of our previously published studies (Jóhannesson et al., 2016, 2017; Á. Kristjánsson et al., 2014). For ease of access we have collated this data and made it available on the OSF page associated with the main text of the current paper at: https://osf.io/6bge9/

Data from 64 participants who completed the desktop version of the task were obtained from the OSF page associated with Clarke et al at: https://osf.io/y6qbv/

Data Selection & Characteristics

From each data set, we selected the first 10 trials of the Feature condition in which each participant correctly cancelled all 40 target items. This gave us 400 responses per observer and 25,600 total samples for each data set. The basic dependent variable was the inter-target time (ITT), calculated by subtracting response N from response N-1 (T. Kristjánsson et al., 2018, 2020), a measure directly comparable to MILO SRTs (Horowitz & Thornton, 2008; Thornton & Horowitz, 2004). As the initial response in each trial appears to have a special status (Basoudan et al., 2019; T. Kristjánsson et al., 2020; Thornton & Horowitz, 2004) these were not included in the analysis. From the remaining 24,960 samples, we further excluded any ITT less than 100 ms or greater than 2000 ms. These exclusions removed 39 samples (0.16%) from the iPad data and 109 samples (0.44%) from the desktop data, respectively.

Reaction Time Distributions

Figure S2 presents the frequency distribution, for both data sets, grouped into 50 ms bins. Consistent with the MILO comparison above, iPad responses appear to have been substantially faster than those made in the desktop study via the mouse (similar to Germine et al., 2019). In addition to shifting the response distributions down, the iPad data are more clearly skewed and have a more prominent peak response (see Table S1 for details). To directly compare performance in terms of latency and precision, we estimated the relevant parameters separately for each observer. As expected from the overall pattern, participants consistently responded more rapidly on the iPad (M = 342 ms, SE = 10) than on the desktop (M = 673 ms, SE = 13) version of the task, t(116.8) = 20.7, p < .001, d = 3.7. The individual response distributions were also less variable on the iPad (SEM = 8.5 ms, SE = 0.3) than on desktop (M = 12.8 ms, SE = 2.1) version of the task, t(122.9) = 10.6, p < .001, d = 1.9.

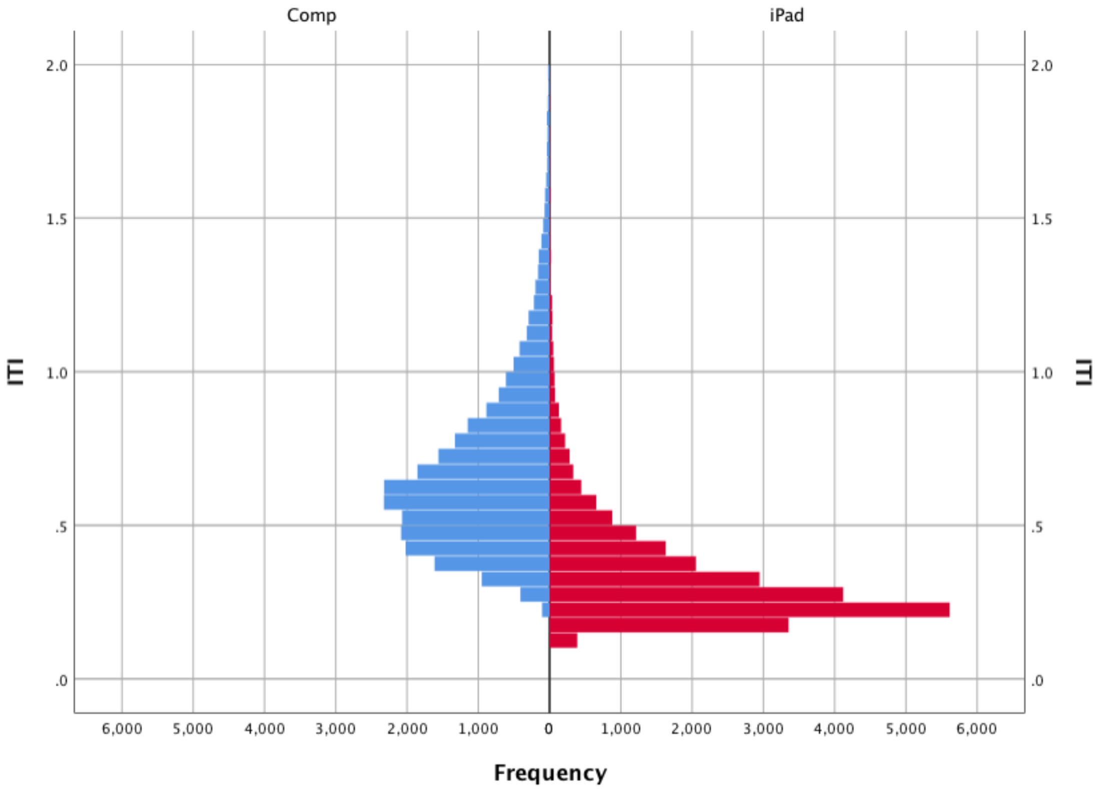

Figure S2. Comparison between response distributions for computer (left) and iPad (right) versions of the human foraging task.

Summary & Conclusions

The goal of this supplementary report was to examine whether there is any evidence that response times collected via an iPad are likely to reduce the sensitivity of the MILO Mobile app relative to the desktop computer implementation used in previous MILO studies. We found that the MILO Mobile app was able to reproduce the patterns observed in previous experiments. Furthermore, we found that the mobile app platform yielded faster response times and reduced variability, relative to desktop computer experiments.

Table S1. Summary of the frequency distributions for both input modalities during the foraging task.

Measure	iPad	Computer
Sample Size	24,921	24,815
Mean (ms)	343	672
Median (ms)	283	617
Mode (ms)	250	616
SEM (ms)	1	2
SD (ms)	190	275
Skewness	2.6	1.3
Kurtosis	10.5	2.4

References

Basoudan, N., Torrens-Burton, A., Jenkins, A., Thornton, I. M., Hanley, C., Tree, J. J., Thomas, S., & Tales, A. (2019). Sequential Information Processing: The "Elevated First Response Effect" Can Contribute to Exaggerated Intra-Individual Variability in Older Adults. *The Yale Journal of Biology and Medicine*, *92*(1), 13–20.

Brainard, D. H. (1997). The Psychophysics Toolbox. *Spatial Vision*, *10*, 433–436.

Brand, A., & Bradley, M. T. (2012). Assessing the Effects of Technical Variance on the Statistical Outcomes of Web Experiments Measuring Response Times. *Social Science Computer Review*, *30*(3), 350–357. https://doi.org/10.1177/0894439311415604

Bridges, D., Pitiot, A., MacAskill, M. R., & Peirce, J. (2020). The timing mega-study: comparing a range of experiment generators, both lab-based and online. *PsyArXiv Preprint*. https://doi.org/https://doi.org/10.31234/osf.io/d6nu5

Burke, D., Linder, S., Hirsch, J., Dey, T., Kana, D., Ringenbach, S., Schindler, D., & Alberts, J. (2017). Characterizing Information Processing With a Mobile Device: Measurement of Simple and Choice Reaction Time. *Assessment*, *24*(7), 885–895. https://doi.org/10.1177/1073191116633752

Clarke, A. D. F., Irons, J., James, W., Leber, A. B., & Hunt, A. R. (2018). *Stable individual differences in strategies within, but not between, visual search tasks*. https://doi.org/10.31234/osf.io/bqa5v

Germine, L., Reinecke, K., & Chaytor, N. S. (2019). Digital neuropsychology: Challenges and opportunities at the intersection of science and software. *The Clinical Neuropsychologist*, *33*(2), 271–286. https://doi.org/10.1080/13854046.2018.1535662

Horowitz, T. S., & Thornton, I. M. (2008). Objects or locations in vision for action? Evidence from the MILO task. *Visual Cognition*, *16*(4), 486–513. https://doi.org/10.1080/13506280601087356

Jóhannesson, Ó. I., Kristjánsson, Á., & Thornton, I. M. (2017). Are foraging patterns in humans related to working memory and inhibitory control? *Japanese Psychological Research*, *59*(2), 152–166.

Jóhannesson, Ó. I., Thornton, I. M., Smith, I. J., Chetverikov, A., & Kristjánsson, Á. (2016). Visual Foraging With Fingers and Eye Gaze. *I-Perception*, *7*(2). https://doi.org/10.1177/2041669516637279

Kleiner, M., Brainard, D. H., Pelli, D. G., Ingling, A., Murray, R., Broussard, C., & others. (2007). What's new in Psychtoolbox-3. *Perception*, *36*(14), 1.

Kristjánsson, Á., Jóhannesson, Ó. I., & Thornton, I. M. (2014). Common Attentional Constraints in Visual Foraging. *PLoS ONE*, *9*(6), e100752. https://doi.org/10.1371/journal.pone.0100752

Kristjánsson, T., Thornton, I. M., Chetverikov, A., & Kristjánsson, Á. (2020). Dynamics of visual attention revealed in foraging tasks. *Cognition*, *194*, 104032. https://doi.org/10.1016/j.cognition.2019.104032

Kristjánsson, T., Thornton, I. M., & Kristjánsson, Á. (2018). Time limits during visual foraging reveal flexible working memory templates. *Journal of Experimental Psychology: Human Perception and Performance*, *44*(6), 827.

Luu, D. (2017). *Computer latency: 1977-2017*. https://danluu.com/input-lag/

Miller, R., Schmidt, K., Kirschbaum, C., & Enge, S. (2018). Comparability, stability, and reliability of internet-based mental chronometry in domestic and laboratory settings. *Behavior Research Methods*, *50*(4), 1345–1358. https://doi.org/10.3758/s13428-018-1036-5

Neath, I., Earle, A., Hallett, D., & Surprenant, A. M. (2011). Response time accuracy in Apple Macintosh computers. *Behavior Research Methods*, *43*(2), 353–362. https://doi.org/10.3758/s13428-011-0069-9

Paul, I. (2013, October 9). *Apple rules, Android drools for responsive tablet touchscreens*. PCWorld. https://www.pcworld.com/article/2053306/apple-rules-android-drools-for-responsive-tablet-touchscreens.html

Pelli, D. G. (1997). The VideoToolbox software for visual psychophysics: transforming numbers into movies. *Spatial Vision*, *10*(4), 437–442.

Plant, R. R. (2016). A reminder on millisecond timing accuracy and potential replication failure in computer-based psychology experiments: An open letter. *Behavior Research Methods*, *48*(1), 408–411. https://doi.org/10.3758/s13428-015-0577-0

Plant, R. R., & Quinlan, P. T. (2013). Could millisecond timing errors in commonly used equipment be a cause of replication failure in some neuroscience studies? *Cognitive, Affective, & Behavioral Neuroscience*, *13*(3), 598–614. https://doi.org/10.3758/s13415-013-0166-6

Pronk, T., Wiers, R. W., Molenkamp, B., & Murre, J. (2019). Mental chronometry in the pocket? Timing accuracy of web applications on touchscreen and keyboard devices. *Behavior Research Methods*. https://doi.org/10.3758/s13428-019-01321-2

Reimers, S., & Stewart, N. (2007). Adobe Flash as a medium for online experimentation: A test of reaction time measurement capabilities. *Behavior Research Methods*, *39*(3), 365–370. https://doi.org/10.3758/BF03193004

Reitan, R. M. (1958). Validity of the Trail Making Test as an Indicator of Organic Brain Damage. *Perceptual and Motor Skills*, *8*(3), 271–276. https://doi.org/10.2466/pms.1958.8.3.271

Schatz, P., Ybarra, V., & Leitner, D. (2015). Validating the Accuracy of Reaction Time Assessment on Computer-Based Tablet Devices. *Assessment*, *22*(4), 405–410. https://doi.org/10.1177/1073191114566622

Thornton, I. M., Bülthoff, H. H., Horowitz, T. S., Rynning, A., & Lee, S.-W. (2014). Interactive Multiple Object Tracking (iMOT). *PloS One*, *9*(2), e86974.

Thornton, I. M., & Horowitz, T. S. (2004). The multi-item localization (MILO) task: Measuring the spatiotemporal context of vision for action. *Perception & Psychophysics*, *66*(1), 38–50. https://doi.org/10.3758/BF03194859

Thornton, I. M., & Horowitz, T. S. (2015). Does action disrupt Multiple Object Tracking (MOT)? *Psihologija*, *48*(3), 289–301. https://doi.org/10.2298/PSI1503289T

Ulrich, R., & Giray, M. (1989). Time resolution of clocks: Effects on reaction time measurement-Good news for bad clocks. *British Journal of Mathematical and Statistical Psychology*, *42*(1), 1–12. https://doi.org/10.1111/j.2044-8317.1989.tb01111.x
